# Lung Worms in Sheep

**Published:** 1887-01

**Authors:** N. S. Townsend

**Affiliations:** Professor of Agriculture, State University, Columbus, Ohio


### Art. VII.—LUNG WORMS IN SHEEP.

BY N. S. TOWNSEND, M. D.
*Professor of Agriculture, State University, Columbus, Ohio.*

My object in sending to you a short paper upon lung worms in sheep is not so much to state what is known as to suggest enquiry in regard to what perhaps is not known. In Ohio about five millions of sheep are usually kept; and next to dogs by far their worst enemy is the lung worm, *Strongylus filaria.* From this cause hundreds of thousands of sheep have died in Ohio in a single season, while scarcely a year passes without the loss of many thousands. The disease occasioned by these parasites is known among farmers here as paper skin, white skin, pelt rot, &c., and among veterinarians as verminous bronchitis, parasitic anæmia, &c.

The early stages of the disease are characterized by fits of coughing and sneezing, with discharge of mucous from the nostrils. The affected sheep stretches the neck, rubs the nose upon the grass, and gives other evidence of difficult breathing, or irritation of the air passages. After a time the animal loses flesh and strength, the eyes and mucous membranes become unusally pale, a diarrhœa comes on and rapidly hastens the general emaciation. The wool becomes loose, and is easily pulled off, or comes off spontaneously in patches, leaving the skin peculiarly bloodless and pale, and hence the popular names, white skin, paper skin, &c. Finally the sheep dies from exhaustion, except in a few cases where in the earlier stages of the disease the smaller bronchial tubes are literally filled with the worms, and the sheep dies of suffocation. Of the Strongylus filaria, it is easy to distinguish the sex, the females being white, and sometimes as much as four inches in length; the males are shorter, and slightly yellow in color; lambs during their first year are most subject to this disease though older sheep are not exempt.

Treatment is usually both dietetic and medicinal; when sheep are fed a liberal allowance of oats or oil cake their powers of resistance to the disease are greatly increased; medical treatment consist of fumigations with sulphur, tar vapor, or chlorine gas; the most effectual probably being the sulphur. The sheep are driven into a close stable, a pan of burning coals is carried in, then the doors and other openings are closed, and either roll brimstone or flowers of sulphur is thrown upon the fire until the fumes of sulphurous acid gas fill the place. The operator should submit to the fumigation with the sheep so that he may be able to judge when they can bear it no longer. Ten or fifteen minutes at a time, and repeated once or twice after intervals of a week, may be sufficient. After fumigation a tablespoonful or thereabouts of a mixture of castor oil and spirits of turpentine, in the proportion of three of the former to one of the latter, should be given to each sheep daily for a few times. When on examination of sheep that have died the worms are found exclusively in the lungs, whisky, linseed oil, or milk may be substituted for the castor oil, and in the same proportion. If in addition to worms in the lungs, the Strongylus rufescens or S. contortus are found in the third or fourth stomach or intestines, the castor oil with the turpentine is preferable. This treatment if skillfully managed is tolerably successful; in April last a farmer in an adjoining county, after fumigating a lot of very sick sheep with sulphur, then gave the castor oil and turpentine; the sheep immediately improved with the exception of one which had become too weak, so that after the fumigation and two doses of the turpentine mixture it died and was sent to the writer for examination. The body of the sheep was found to be completely anæmic, the lungs had considerably more than their natural density, the result of inflammatory exudation; but after the most diligent search, not a single worm could be found in any of the bronchial ramifications, while in the nostrils, and attached to the teeth were several dead worms, the last that had been coughed up before the sheep had ceased to breathe.

But inasmuch as prevention is better than cure, a complete life history of the parasite is greatly needed, so that it may be avoided or destroyed before its mischief is done. Cobbold* says "the question which agriculturists should be chiefly desirous of solving is that appertaining to the mode of origination and development of these parasites, for by acquiring accurate data on this head, it is not altogether unreasonable to suppose that we may hit upon a more or less perfect method of limiting or eradicating the disastrous epizootics associated with an excessive multiplication of these creatures during particular seasons." Van Beneden† says of nematode or threadlike worms, "that about a thousand varieties are known, varying in length from a few millimeters to forty or fifty centimeters. They are not all parasitic, since some are found in the sea, and others in damp earth, in putrid matter, and even upon plants. The migrations of nematodes are subjects of great interest; their changes of form are not usually very considerable, but the modifications in their sexual apparatus, whether in the same individual or in succeeding generations, are very curious. When we consider the numerous encysted, and agamous nematodes which are found in the different orders of mammalia, birds, reptiles, batrachians, and fishes, there is little doubt that all these beings are only migratory parasites which pass together with their hosts into the animals to which they are destined. They are found, like ascarides, in animals of all classes; some are to be met with in all the organs, the brain, the eye, the muscles, the heart, the lungs, the tracheal artery, the frontal sinus, the digestive tube, the skin, and even in the blood. Sometimes the two sexes live under the same conditions, sometimes the male is dependent on its female, or else one generation is parasitic and the next is independent. There is great diversity with respect to de-

*Internal Parasites of the Domestic Animals.

†Animal Parasites and Messmates.

velopment; some nematodes like trichinæ are developed so rapidly that the embryo is already perfect in the egg before it has quitted its mother; others like the Ascaris lumbricoides lay eggs in which the embryos do not appear for several weeks or even months after they have been laid. Between these two extremes we find all the intermediate degrees." That Strongylus filaria is able to maintain an independent life in damp earth for a time is no longer a question. Professor Ercolani obtained these parasites from the lungs of a goat, kept them alive in damp earth, saw them reproduce, and was able to obtain several generations of them. But that they have other than mammalian host is equally certain, and that these may have much to do with their occasional superabundance is more than probable. Orthopterous and Coleopterous insects, are often found infested with gordii; common mollusks, such as Limax agrestis, harbor the Distoma hepaticum or liver fluke; and there is reason to suspect that various aquatic mollusks such as Limnæus and Physæ or Planorbis, &c., may be hosts of strongyli, as fish are well known to be. Should these nematodes be abundantly found in our fresh water mollusks or their embryos found in running water, it might explain the special danger to which sheep are exposed by pasturing on meadows which have been subject to overflow. In Europe as many as eight or nine species of strongyli infesting different animals are known; in this country, it is not unusual to find three species in the lungs or intestinal canal of the same sheep.

If zoology were a more popular study among farmers, or if veterinarians would more frequently give special attention to the entozoon of our domestic animals, is it not probable that in localities where lung worm disease is prevalent, important discoveries in regard to the life history of the parasite might be made, and its ravages in future measurably prevented?

